# Deep Brain Stimulation for Chronic Facial Pain: An Individual Participant Data (IPD) Meta-Analysis

**DOI:** 10.3390/brainsci13030492

**Published:** 2023-03-14

**Authors:** Hebatallah Qassim, Yining Zhao, Armin Ströbel, Martin Regensburger, Michael Buchfelder, Daniela Souza de Oliveira, Alessandro Del Vecchio, Thomas Kinfe

**Affiliations:** 1 Department of Neurosurgery, Friedrich-Alexander University (FAU) Erlangen-Nürnberg, 91054 Erlangen, Germany; 2 Center for Clinical Studies (CCS), Medical Faculty, Friedrich-Alexander University (FAU) Erlangen-Nürnberg, 91054 Erlangen, Germany; 3 Division of Molecular Neurology, Friedrich-Alexander University (FAU) Erlangen-Nürnberg, 91054 Erlangen, Germany; 4 Department of Artificial Intelligence in Biomedical Engineering (AIBE), Friedrich-Alexander University (FAU) Erlangen-Nürnberg, 91054 Erlangen, Germany; 5 Division of Functional Neurosurgery and Stereotaxy, Department of Neurosurgery, Friedrich-Alexander University (FAU) Erlangen-Nürnberg, 91054 Erlangen, Germany

**Keywords:** facial pain, deep brain stimulation, efficacy, safety, predictive outcome measures, placebo

## Abstract

Despite available, advanced pharmacological and behavioral therapies, refractory chronic facial pain of different origins still poses a therapeutic challenge. In circumstances where there is insufficient responsiveness to pharmacological/behavioral therapies, deep brain stimulation should be considered as a potential effective treatment option. We performed an individual participant data (IPD) meta-analysis including searches on PubMed, Embase, and the Cochrane Library (2000–2022). The primary endpoint was the change in pain intensity (visual analogue scale; VAS) at a defined time-point of ≤3 months post-DBS. In addition, correlation and regression analyses were performed to identify predictive markers (age, duration of pain, frequency, amplitude, intensity, contact configuration, and the DBS target). A total of seven trials consisting of 54 screened patients met the inclusion criteria. DBS significantly reduced the pain levels after 3 months without being related to a specific DBS target, age, contact configuration, stimulation intensity, frequency, amplitude, or chronic pain duration. Adverse events were an infection or lead fracture (19%), stimulation-induced side effects (7%), and three deaths (unrelated to DBS—from cancer progression or a second stroke). Although comparable long-term data are lacking, the current published data indicate that DBS (thalamic and PVG/PAG) effectively suppresses facial pain in the short-term. However, the low-quality evidence, reporting bias, and placebo effects must be considered in future randomized-controlled DBS trials for facial pain.

## 1. Introduction

Chronic pain represents an enormous burden for affected persons along with an impaired quality of life. Approximately 20–30% of the population in modern industrialized countries suffer from chronic pain of various origins. With a share of 26%, facial pain is one of the most common types of chronic pain in Western industrialized countries [1,2,3]. Despite the distinct differences in the pathophysiology of facial pain syndromes, first-line therapies such as pharmacotherapies, behavioral therapies and less invasive neurostimulation approaches (peripheral nerve stimulation and occipital nerve stimulation) fail to achieve a sustained and meaningful responsiveness [4,5,6,7,8]. Depending on the type, additional functional therapies may be used. As a more invasive procedure, the use of motor cortex stimulation (MCS) has been demonstrated as an effective treatment option for chronic facial pain [9,10]. In cases where there is a limited responsiveness to MCS, deep brain stimulation (DBS) represents an additional, although more invasive, treatment option for chronic facial pain; however, negative results stemming from two randomized-controlled trials at the end of the last century indicated a negative treatment outcome. Previous studies have described different DBS targets, depending on the pain syndrome. The central median nucleus/parafascicular complex (Cm-Pf), the nucleus ventralis posteromedialis (VPM) and the nucleus ventralis posterolateralis (VPL) of the thalamus, as well as the periventricular/peri-aqueduct grey of the brainstem (PVG/PAG), have been targeted in various studies in humans [11,12,13,14,15,16,17,18,19,20,21,22,23,24,25,26,27].

However, observational cohort studies, case series, and/or small-scale uncontrolled trials have provided low-level evidence for the use of DBS in the treatment of facial pain syndromes due to the heterogeneity of the data published with respect to the DBS target, stimulation patterns, facial pain etiology, hardware-/stimulation-associated adverse events, and the observation period post-DBS [28,29].

Hence, open questions remain which have been a matter of ongoing debate and recommendations. We, therefore, performed a meta-analysis of individual participant data (IPD) of patients that were treated with deep brain stimulation for chronic facial pain and addressed the aforementioned questions.

## 2. Materials and Methods

### 2.1. Search Design and Data Focus

This IPD-based meta-analysis was designed according to the PRISMA framework for an individual participant data (IPD)-based analysis because an aggregated data analysis is hindered due to the low numbers of randomized-controlled trials. We conceptualized the review purpose in line with the PICO (P: patients; I: interventions; C: comparison; O: outcome) guidelines for chronic facial patients (P), who were treated with DBS (I) along with the assessment of potential predictive factors (C) that were associated with DBS responsiveness (O). For this purpose, we conducted a structured literature search covering the period 2000–2022. We used searches on PubMed, Ovid MEDLINE, Embase, the Cochrane Library, and Scopus. The search terms that were used here included “facial pain”, “trigeminal neuralgia”, “pain relief”, “deep brain stimulation”, “thalamic stimulation”, periventricular/peri-aqueduct grey”, “safety”, “efficacy”, and “adverse events”.

### 2.2. Inclusion and Exclusion Criteria

A manual library search was performed by three independent reviewers (MN, YZ, and TK) following defined inclusion and exclusion criteria for data sampling, as follows. The inclusion criteria were DBS in human studies for refractory facial pain (case series, cohort studies, and uncontrolled/controlled trials) reporting score-based outcome measures (visual analogue scale, numeric rating scale, and percentage reduction); stimulation parameters (mono-polar/bipolar, frequency, amplitude, and intensity); demographics (age, gender, and facial pain duration); and DBS targets. The exclusion criteria were the use of other non-invasive and invasive neuromodulation techniques (motor cortex stimulation and peripheral nerve stimulation). DBS studies targeting the thalamus and the PVG/PAG were considered, as most of the data points were available for these targets. As pharmacotherapy was inconsistently reported over the course of the DBS, changes in medication were not included in the meta-analysis.

A variety of diagnoses were found classified as chronic facial pain in the literature and included facial pain originating after a stroke, trigeminal neuropathy/neuralgia, post-infection facial pain, post-surgical facial pain, and facial pain derived from lesions in the trigeminal-nociceptive system. Furthermore, it is noteworthy that all the IPD data were derived from the published literature only, and we did not approach the investigators of the included studies to seek data reporting negative, unpublished results.

### 2.3. Definitions of Primary/Secondary Outcome Measures and the Additional Assessment of Possible Relationships between DBS Outcomes versus Stimulation Parameters and Patient Characteristics

The primary endpoint was defined as a change in pain intensity quantified by changes in scores (reduction and/or percentage) post-DBS treatment. In most of the assessed literature, comparable data were only available post-DBS, defined as ≤3 months post-implantation and set as the time-point of the presented meta-analysis. Longer follow-up timeframes were reported in fewer than five studies. The stability and reliability of our IPD two-stage analytic approach was limited by the inconsistent determination in the extracted studies, of factors such as the sleep quality, mood, cognitive psychometrics, and functional capacities, as relevant, and, frequently, the co-deteriorated clinical characteristics of chronic facial pain and the potential markers of secondary outcomes. No sufficient DBS “On” versus “Off” data were available. Thus, the findings that are provided herein must be considered as short-term, covering the first 3 months post-DBS treatment. In addition, to extrapolate the possible negative and positive predictive measures for DBS responsiveness, correlative statistics were applied targeting age, contact configuration, intensity, frequency, amplitude, pain duration, and the DBS target (thalamic nuclei versus PVG/PAG versus combined thalamic + PVG/PAG).

### 2.4. Statistical Analysis

The patient demographics are displayed by the mean value and standard deviation or the relative numbers and percentages. The outcomes and results of the included studies are displayed by their mean values and standard deviations. Due to the heterogeneous data structure of the included studies, the aggregated data analysis was limited. Therefore, a meta-analysis of individual participant data (IPD) was performed. Various approaches for conducting IPD meta-analyses (IPD-MA) have been developed in recent years. In the context of this study, an evaluation was performed using a mixed effects model [29]. We additionally calculated other IPD synthesis models with random effects, described in Debray (2015), but these models did not converge. Other studies were not further analyzed as they did not report score-based changes in pain intensity.

## 3. Results

### 3.1. Baseline Characteristics and the IPD Data Extraction Protocol

The baseline clinical characteristics of the included subjects for IPD analysis are given in Table 1. As already mentioned, comparable datasets (facial pain etiology, DBS outcomes, stimulation parameters, age, disease duration, adverse events, and medication intake changes) were available across most studies for the short-term follow-up period reported in the published literature (ranging from post-DBS implantation to 3 months). Standardized outcome reports that were feasible for analysis were limited when assessing a follow-up period longer than 3 months. Therefore, the results that are provided in this meta-analysis reflect short-term data.

The mean age was 54 ± 14 years (49% male; 32% female; 19% not reported) and the data included the following chronic facial pain origins: post-stroke, 32%; post-surgical, 22%; trigeminal, 16%; post-infection, 12%; and not reported, 18%. The DBS targets consisted either of thalamic nuclei (CmPf; VPM, VPL) in 40%, PVG/PAG in 31%, and a combined DBS protocol (CmPf; VPM, VPL + PVG/PAG) in 19% of the included studies.

**Figure 1:**
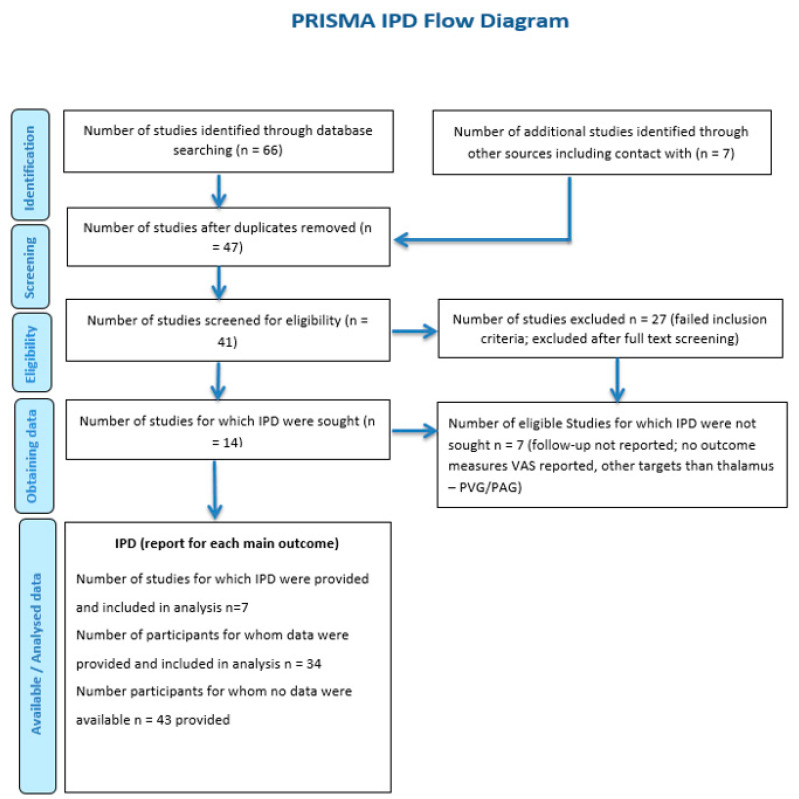
The PRISMA flow-chart of the assessed DBS studies with humans for treatment of facial pain.

There were 34 subjects that were eligible for inclusion as final IPD patients (43 were excluded according to our study protocol); these were derived from seven DBS studies with humans and analyzed at ≤3 months post-DBS. All the reports represent single-arm uncontrolled studies; thus, no blinding or randomization was applied. Data for a longer follow-up period were limited, and the IPD demonstrated instability due to the sparse data (Figure 1).

### 3.2. The Primary Endpoint (a Reduction in PAIN Intensity)

A reduction in pain was quantified by a visual analogue scale (VAS).

**Figure 2:**
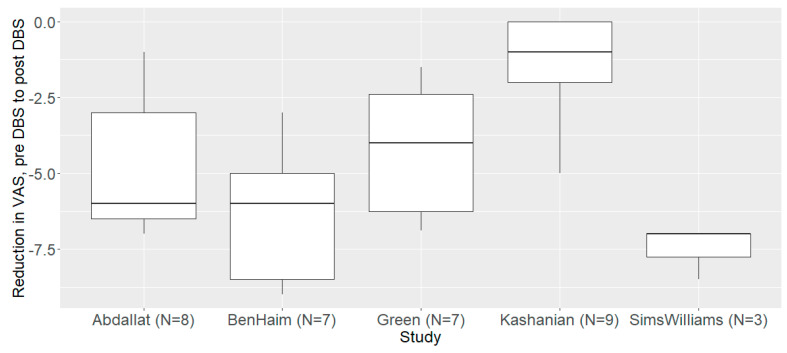
The VAS level changes pre- and post-DBS at 3 months (T1) follow-up for the included studies as per the study definition. The boxplot shows the median, the upper and lower quartiles, and the two whiskers. The upper whisker extends from the upper quartile to the largest value no further than 1.5 × IQR from the upper quartile, where IQR is the inter-quartile range, or distance between the upper and lower quartiles. The lower whisker has an analogous definition.

We analyzed those studies reporting changes in the VAS levels pre- and post-DBS and those reporting percentage changes in the VAS separately. For a reduction in the VAS, the synthesis of individual participant data was carried out by a linear model with the equation VAS ~ 0 + Source + time. The overall reduction in the VAS was 4.6 points, see Table 2. These findings were confirmed when specifically assessing the included participants for each study [(Abdallat et al. [12]: *p* < 0.001; pain reduction = −4.7; st.error = 0.86; t = −5.5); (Ben Haim et al. [15]: *p* <0.001; pain reduction = −6.4; st.error = 0.86; t = −7.4); (Green et al. [19]: *p* <0.001; pain reduction = −4.2; st.error = 0.86; t = −4.9); (Kashanian et al. [20]: *p* <0.001; pain reduction = −1.6; st.error = 1.02; t =−1.6); (Sims Williams et al. [25]: *p* <0.001; pain reduction = −7.5; st.error = 1.32; t = −5.7)]; (ANOVA: *p* = 0.007; df = 4; sumsq = 94; meansq = 23.5; f = 4.5) (Figure 2/Table 2).

**Figure 3:**
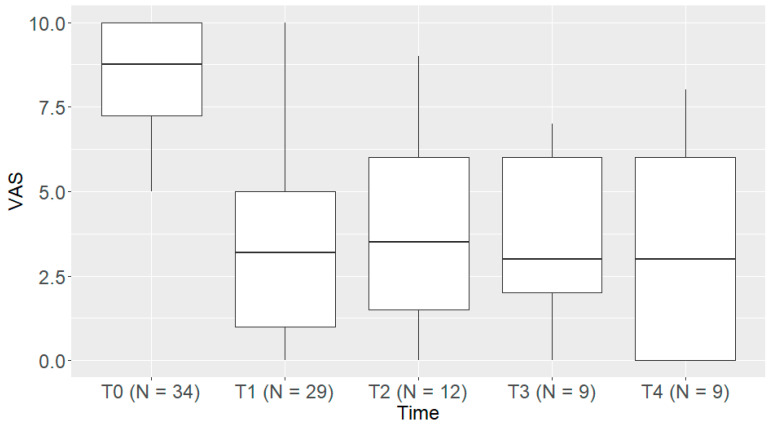
The VAS level changes pre- and post-DBS and numbers of patients when longer follow-up periods were considered: T0 (baseline), T1 (3 months), T2 (12–24 months), T3 (24–47 months), and T4 (≥48 months). The boxplot shows the median, the upper and lower quartiles, and the two whiskers. The upper whisker extends from the upper quartile to the largest value no further than 1.5 × IQR from the upper quartile, where IQR is the inter-quartile range, or distance between the upper and lower quartiles. The lower whisker has an analogous definition.

Data that were derived from the follow-up period beyond 3 months (defined as T1) were not eligible for further analysis; therefore, only the VAS reduction was analyzed for 12 participants at T2 and for 9 patients at T3 and T4. We found a significant VAS reduction across all of the included follow-up periods. Notably, these data were extracted from one study [12] (*p* < 0.001) (Figure 3).

**Figure 4:**
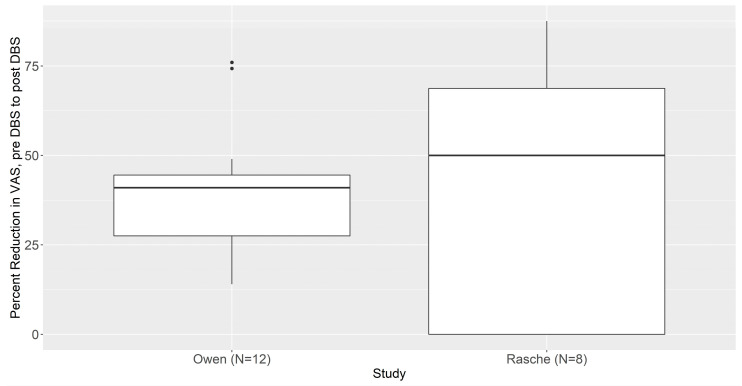
The percentage change in pain scores pre- and post-DBS at 3 months follow-up for the included studies. The boxplot shows the median, the upper and lower quartiles, and the two whiskers. The upper whisker extends from the upper quartile to the largest value no further than 1.5 × IQR from the upper quartile, where IQR is the inter-quartile range, or distance between the upper and lower quartiles. The lower whisker has an analogous definition (this is Tukey’s definition of a Boxplot).

For the percentage reduction in the VAS, the synthesis of individual participant data was performed by a mixed effects model with the model equation VAS∼ 1 + (1|Study). The overall percentage reduction in the VAS in this model was 41% (std.error = 6.24, t-value = 6.56, *p*-value =< 0.001). According to our protocol, we identified two studies fulfilling the criteria [(Owen et al. [23]: *p* < 0.001; pain reduction = 40; st.error = 8.3; t = 4.8); (Rasche et al. [13]: *p* <0.001; pain reduction = 42; st.error = 10.1; t = 4.2)]; (ANOVA: *p* = 0.870; df = 1; sumsq = 22; meansq = 22; f = 0.027) (Figure 4).

### 3.3. The Subgroup Analysis between PVG/PAG and Thalamic DBS (VPL/VPM/CmPf)

**Figure 5:**
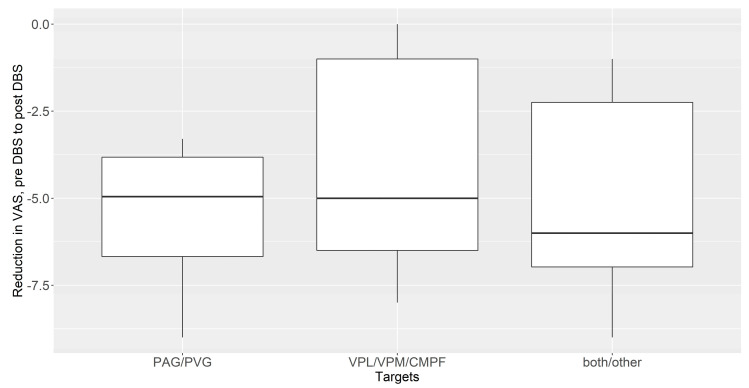
The subgroup analysis analyzing DBS outcomes depending on the choice of the target between PVG/PAG alone and thalamic DBS alone (VPL/VPM/CmPf) versus combined targeting. The boxplot shows the median, the upper and lower quartiles, and the two whiskers. The upper whisker extends from the upper quartile to the largest value no further than 1.5 × IQR from the upper quartile, where IQR is the inter-quartile range, or the distance between the upper and lower quartiles. The lower whisker has an analogous definition (this is Tukey’s definition of a Boxplot).

With regard to the choice of the DBS target, we found no significant differences when comparing the thalamic nuclei versus the PVG/PAG and/or the combined approach targeting both the PAG/PVG (PAG/PVG versus VPL/VPM/CmPf: *p* = 0.42; t = −0.82; df = 26; std.error = 1.7; pain reduction = −1.37); (PAG/PVG versus others: *p* = 0.74; t = −0.34; df = 26; std.error = 1.6; pain reduction = −0.55); (VPL/VPM/CmPf versus others: *p* = 0.48; t = 0.71; df = 26; std.error = 1.2; pain reduction = 0.82) (Figure 5/Table 3).

### 3.4. The Assessment of Correlations between the DBS Outcome and Age, Stimulation Protocol, Facial Pain Duration, and the DBS Target (Spearman’s Correlation)

To investigate predictive outcome measures for DBS, we analyzed the potential relationship between pain intensity changes and the following features: age, contact configuration (monopolar versus bipolar), intensity, frequency, amplitude, duration of facial pain, and DBS target (thalamus versus PVG/PAG). Correlation assessment demonstrated no relationship between DBS response (pain reduction) and age (r = −0.049; *p* = 0.80), contact configuration (*p* = 0.29), stimulation intensity (r = −0.004; *p* = 0.982), frequency (r = 0.037; *p* = 0.85), and amplitude (r = 0.196; *p* = 0.308), despite with the duration of the underlying facial pain etiology (r = −0.389; *p* = 0.03).

Notably, to detect a correlation of 0.4 as significant, more than 46 observations are needed (alpha = 5%, power = 80%, two-sided test, correlation in a bivariate normal model, Software GPower 3.1.9.7). Hence, the statistical tests of our correlation analysis were underpowered.

### 3.5. Adverse Events

In general, hardware-associated adverse events and stimulation-related side effects have been reported inconsistently and have occurred mainly after ≥6 months post-DBS in the analyzed studies. Overall, the incidence of severe adverse events was low in the first 3 months. Infection and lead fracture were observed in 10/54 (19%) cases and stimulation-evoked adverse events were noticed temporarily and resolved after re-programming. However, stimulation-induced side effects were recorded in 5% (3/54) of cases, along with three deaths (7%), which were unrelated to DBS and due to cancer progression and the occurrence of a second stroke.

## 4. Discussion

### 4.1. General Remarks on the Use of DBS for Refractory Chronic Facial Pain

Despite its “Off Label” character due to failures in the two RCT studies for FDA approval and the low-to-moderate evidence, DBS that is targeting either the nuclei of the thalamus, relevant to sensory (VPL) and affective (CmPf, CLT) pain perception and processing alone and/or in combination with the brainstem-associated pain circuits (PVG/PAG) that are capable of modulating opioid pathways, has effectively and safely demonstrated the promotion of clinically meaningful responsiveness for chronic pain disorders. This includes, but is not limited to, facial pain (trigeminal-nociceptive system) in a series of uncontrolled human studies [30]. This failure of FDA approval has stemmed from considerable differences in trial protocol, calculation of efficacy rates, prolonged and slow enrollment, variabilities of pain etiologies enrolled (nociceptive versus neuropathic), pain diagnosis, follow-up period, hardware dysfunction, and the DBS targets (the VPL/VPM nuclei of the thalamus and the PAG/PVG region of the brainstem). Furthermore, the DBS response was defined as at least 50% pain reduction after 1 year in half of the participants. In addition, different DBS leads were applied, of which one model (Medtronic 3380) was no longer available from the manufacturing company [30].

Although most of the published studies with humans have reported promising results, the heterogeneity of study protocols, the availability of less-invasive neurostimulation techniques (e.g., motor cortex stimulation and peripheral nerve/nerve field stimulation), along with the lack of imbursement represent considerable barriers for the application of DBS for chronic facial pain. While these findings were not validated by high-quality evidence, this may not limit the value of the use of DBS in general and the authors advocate the general integration and the therapeutic potential of cerebral neurostimulation where less-invasive approaches have failed.

### 4.2. The Impact of DBS on Facial Pain and Functional Capacity

In line with previously published DBS studies with humans, our results indicate that DBS controls chronic facial pain in a safe manner, although limited long-term homogenous data exist. Therefore, our findings cover a short-term follow-up period (3 months). Whilst these effects were observed across different DBS targets (VPL/VPM; CmPf; PAG/PVG), the clear superiority of a specific target was not present, nor did we find correlations between age, gender, facial pain disease duration, stimulation parameters, and DBS outcomes. Adverse events occurred in a relatively low proportion of DBS patients and were resolved in most cases. However, one should bear in mind our short-term observation period of 3 months from DBS therapy. Notably, many of the assessed facial pain patients were implanted with a combination of thalamic and brainstem nuclei, limiting an unbiased comparison. In addition, some small-scale studies have determined novel DBS targets, such as ACC and/or ALIC/VS, primarily modulating the affective brain circuits that are relevant to neural pain perception and processing [31].

It is important to understand that chronic pain (this holds true for facial pain as well) reflects a multidimensional disorder of the brain affecting several distinct brain networks that are relevant to the neurophysiological processing of nociceptive stimuli. De Ridder et al. revisited the framework that was initially proposed by Melzack and co-workers, discussing a refined definition and framework for chronic pain, thus distinguishing the different networks that are involved in sensory, affective–cognitive, and descending pain neural transmission. This differentiation is of great importance for neurostimulation therapies, with the potential to identify novel DBS targets and the corresponding stimulation patterns. Furthermore, the authors point to the emerging and unresolved issues of whether a chronic patient is more or less likely to respond to sensory, affective, and/or cognitive-focused neuromodulation and advocates for the selective characterization of the brain’s pain matrix, which in turn may help to identify suitable targets and patients. Brain stimulation techniques that selectively target the affective–cognitive pathways for chronic pain have been applied in a growing number of pain patients, considering the multidimensional characteristics of the brain’s pain matrix. These strategies have been supported by neuroimaging studies assessing structural and functional changes in pain-associated networks. Therefore, it is not surprising, that different DBS approaches (sensory versus affective modulation) may impact facial pain with similar favorable outcomes, although the mechanism of action may differ [32].

### 4.3. A Brief Discussion of Long-Term DBS Studies and Alternative DBS Targets (ACC; ALIC/VS)

Although not fulfilling the inclusion criteria for our IPD-based analysis, it is worth briefly reflecting on and discussing the data that were derived from long-term DBS trials (uncontrolled) covering DBS outcomes over a period of up to 5 years [12,15,33].

Krauss and co-workers determined DBS outcomes in a variety of chronic pain disorders assessing a large cohort of 40 patients, of whom 10 patients suffered from chronic refractory facial pain (post-surgical, post-infection, post-hemorrhage, and post-ischemia). Of these 10 patients, six patients were treated with CmPf-DBS (affective modulation) and four patients received VPL/VPM-DBS (sensory modulation) with responder rates of 40% (defined as pain reduction ≥ 50%), and even higher responder rates when defining positive outcomes as a pain reduction of ≥ 30%. Similar DBS responder rates were observed in the Oxford DBS unit targeting the PVG/PAG in 39 patients [15,33]. According to a systematic review by Levy et al., thalamic DBS appears to be superior for neuropathic pain, while the PAG/PVG was the DBS target of choice in order to treat nociceptive pain disorders [34]. Lempka et al. conducted an RCT (sham) DBS study in a cohort of 10 post-stroke patients treated with bilateral ALIC/VS DBS, including a randomized phase of 6 months (sham versus verum stimulation) followed by an open label phase of 24 months. The primary endpoint (pain reduction of 50%) was not achieved, although secondary endpoints defined as functional improvements demonstrated positive outcomes of the affective domains of pain. Notably, 1 out of the 10 participants suffered from hemi-facial pain after a stroke. Seizure was observed under DBS “On” as well as under DBS “Off” conditions. Another group determined the impact of bilateral ACC-DBS in five thalamic pain syndrome patients, of whom two suffered from hemi-facial pain. Adverse events were not reported, with a mean pain relief rate of 37% on the numeric rating scale (NRS), which was sustained after 18 months (35% mean NRS reduction). A further observational ACC-DBS pilot study (uncontrolled) was carried out by the Oxford Group in 22 neuropathic pain patients with whole-body/hemi-body pain distribution, and the results showed a mean pain relief of 60% (NRS) after 6 months and 43% NRS decline after 12 months. Notably, the inclusion criteria were defined as failure with VPL/VPM and/or PVG/PAG DBS. Both, the ACC and the ALIC/VS are embedded in the limbic pain circuits of the brain [31].

The ACC contains reciprocal projections with the intra-laminar nuclei of the thalamus (CmPf, CLT), ALIC/VS, the amygdala, the insula, and the dorsolateral prefrontal cortex, while in turn the ALIC/VS regulates orbitofrontal projections to thalamic-cortical circuits relevant to mood and reward behavior [31].

In the case of the failure of lesser or non-invasive strategies, DBS still poses additional challenges with respect to the target. Whilst most published DBS trials for chronic pain disorders report outcomes after thalamic (VPL/VPM; CmPf) and PAG/PVG stimulation, unresolved issues remain concerning whether to modulate the sensory (VPL/VPM) or affective (CmPf; ACC; ALIC/VS) circuits or both. The distinction between sensory or affective DBS responders is not well established. Furthermore, if a decision was rendered to approach the affective networks of the limbic pain matrix by DBS, several targets (CmPf; ACC; ALIC/VS) can and must be discussed. Ongoing DBS studies in Europe and the U.S. currently compare ACC- versus VPL-DBS for chronic pain [31]. Ultimately, the choice of the “gold” DBS target for selective pain disorders remains largely unknown, as there is no reliable guiding biomarker available for DBS in chronic pain [12,31]. The lack of imbursement and the degree of invasiveness should be encountered prior to DBS, and therefore, DBS should be considered after other non-invasive and less-invasive alternative neurostimulation therapies. However, evidence-based robust and comparative studies in favor of, or against, one neurostimulation approach are lacking.

### 4.4. The Evidence Level of Less Invasive and Non-Invasive Neurostimulation Therapies for Chronic Pain

In previous years, a broad variety of less invasive neuromodulation therapies (as compared to DBS) gained attention in interventional pain management [35,36,37,38].

Non-invasive techniques such as repetitive transcranial magnetic stimulation (rTMS), transcranial direct current stimulation (tDCS), transcranial alternating current stimulation (tACS), vagal nerve stimulation (VNS), and transcutaneous electrical nerve/nerve field stimulation (tNFS) have yielded mixed results, displayed by low-quality evidence for tDCS/tACS and tNFS, while the current evidence of moderate quality for rTMS indicates no sustained pain relief. The level of evidence for less invasive spinal cord stimulation (SCS) and peripheral nerve/nerve field stimulation (PNFS) has been classified as low–moderate depending on the pain disorder being treated (e.g., back pain, leg pain, and primary headache disorders). Despite the fact that novel stimulation waveforms were available, thus permitting paraesthesia-free stimulation (e.g., burst SCS, high frequency SCS) and sham (placebo)-controlled study protocols, the majority of published studies with humans have not included sham/control groups. It is of note that some of the therapies that are mentioned are CE marked (SCS and PFNS) and some are not (DBS, MCS, and ONS); therefore, they can be used “off label” [35]. The mechanism of action remains largely unknown for the vast majority of the abovementioned neurostimulation therapies regardless of their differences in the degree of invasiveness. The appropriate patient selection and the pre-stimulation diagnostic uncertainty further increases the likelihood of biased reports in interventional pain management by neuromodulation means. The question of ‘how and when to apply which neurostimulation strategy for whom?’ prevails. Currently, the expert´s opinion and consensus-based guidelines and recommendations appear to be the best solution for this therapeutic dilemma [36]. However, these recommendations are helpful for understanding chronic pain disorders and the related impact of different neurostimulation therapies. For instance, the European Association of Neurology (EAN), the European Federation of Neurological Societies (EFNS), and the Neurostimulation Appropriateness Consensus Committee of the Neuromodulation Society (NACC) have formed interdisciplinary task forces and are conforming the heterogeneous and, in part, inconclusive state of neuromodulation for chronic pain [37,38].

### 4.5. The Impacts of Placebo and Lesion Effects in Deep Brain Stimulation and How to Overcome Them

Biasing factors interacting with the therapeutic DBS effects, observed for movement disorders and chronic pain, represent a matter of ongoing discussion coined as placebo and/or lesion effects caused by the DBS electrode insertion. This holds true for newly developed innovative stimulation technologies, as well as raising considerable expectations for pain relief and functional improvement on both sides (for the patient as well as the physician). Currently, these biasing factors are gaining more and more of the deserved attention and potential solutions are being discussed to counterbalance this mismatch relevant to the interpretation of the observed DBS effects in general. Potential solutions include, but are not limited to, the implementation of novel stimulation protocols with the capability of adaptive stimulation together with the appropriate study protocols which aim to limit the potential biasing issues in future DBS trials [39]. Preliminary results indicate that placebo-derived analgesia may be predicted, and is likely to occur to an increasing degree as a consequence of pre-conditioning. Elaborating and understanding these mechanisms is essential for a more precise strategy in pharmacological as well as neurostimulation treatment and should include neurobiological and psychological characteristics [40,41,42].

### 4.6. Limitations and Prospects

Some aspects of bias in our findings deserve specific attention for several reasons. The data are derived from different neurosurgical centers with different operative DBS protocols (awake versus asleep, intraoperative test-stimulation versus postoperative DBS trial stimulation using externalized leads, intraoperative microelectrode mapping versus direct image-guided DBS lead placement, and the patient and DBS target selection) relevant to the lead contact accuracy and the DBS efficacy, limiting the reproducibility of these data. Despite the fact that we assessed a relatively large proportion of 54 DBS patients suffering from drug-resistant facial pain, a large-scale analysis may have increased the worthiness of our observations. In addition, the data that were assessed were derived mostly from uncontrolled case studies, usually reporting positive outcomes and, therefore, tempering the conclusions. This holds true for the time analyzed in our report (3 months post-DBS) as the DBS outcomes that were reported are biased by the placebo effects at 6 months post-DBS. Hence, controlled trials covering a homogenous follow-up longer than 12 months are highly recommended. Novel DBS targets such as the ACC and/or the ALIC/VS, impacting the limbic pain matrix of the brain, were not included (despite CmPf) in our IPD-based analysis and should be considered in the choice of the DBS target.

## 5. Conclusions

Conclusively, our data demonstrate that DBS effectively suppressed facial pain with a relatively low complication rate in the short-term which is likely impacted by placebo and lesion effects. The interpretation of our findings is limited as most of the data that were assessed in our meta-analysis were derived from uncontrolled case studies. In addition, there was a broad variety of origins of the facial pain disorders that were assessed in our meta-analysis. Hence, long-term controlled (unbiased) studies are urgently needed to generate high-quality evidence for DBS therapies for chronic facial pain.

## Figures and Tables

**Table 1:** Overview of the published data reporting the characteristics of the study participants as relevant to the meta-analysis (age, gender, facial pain etiology, and DBS targets).

Data Extracted Prior to IPD Analysis	
Eligible/Screened	34/77
Age (years)	
Mean (SD)	54 ± 14
Gender	
F	25 (32%)
M	38 (49%)
(missing)	14 (19%)
Facial Pain origin	
Stroke	25 (32%)
Trigeminal.	12 (16%)
Post-infection	9 (12%)
Post-Surgical	17 (22%)
(missing)	14 (18%)
DBS target	
VPL/VPM/CmPF	31 (40%)
PAG/PVG	24 (31%)
Combined	15 (20%)
others	7 (9%)

**Table 2:** IPD synthesis for changes in pain intensity quantified by VAS reduction. Table 2 shows the estimated baseline values of the studies and the estimated overall effect of −4.64 points with a standard error of 0.54 points. Covariate assessment included age, contact configuration, intensity, frequency, amplitude, duration of pain disorder, and DBS target. The reference level for variable “Target” was PVG/PAG.

Study	Estimate	Std.Err.	t Value	*p* Value
Source Abdallat	8.00	4.10	1.95	0.057
Source BenHaim	8.70	2.74	3.18	0.003
Source Green	8.74	2.37	3.68	<0.001
Source Kashanian	11.35	3.95	2.87	0.006
Source Sims Williams	9.03	3.22	2.81	0.007
VAS pre vs. post-DBS	−4.64	0.54	−8.63	<0.001
Age	0.00	0.02	0.09	0.930
Lead_Conf. bipolar	−0.60	1.11	−0.54	0.593
Intensity_max	−0.09	0.33	−0.28	0.780
Freq. max	−0.01	0.02	−0.60	0.548
Amplitud.max	0.00	0.01	0.27	0.789
Facial pain Duration months	0.00	0.01	−0.53	0.600
Target VPL/VPM CmPf	1.04	1.43	0.72	0.474
Targets both/other	0.08	0.92	0.08	0.934

**Table 3:** A comparison of the most frequently applied DBS targets. PVG/PAG alone versus CmPf/VPM/VPL alone versus combined targeting (PVG/PAG + CmPf/VPM/VPL).

DBS Targets	Estimate	SE	df	t Ratio	*p* Value
PAG/PVG vs. VPL/VPM/CmPf	−1.37	1.67	26	−0.82	0.42
PAG/PVG alone vs. both targets	−0.55	1.62	26	−0.34	0.74
VPL/VPM/CmPf alone vs. both targets	0.82	1.15	26	0.71	0.48

## Data Availability

Not applicable.
